# An unwanted companion reaches the country: the first record of the alien mosquito *Aedes japonicus japonicus* (Theobald, 1901) in Slovakia

**DOI:** 10.1186/s13071-021-05062-0

**Published:** 2021-11-12

**Authors:** Viktória Čabanová, Kristína Boršová, Marek Svitok, Jozef Oboňa, Ivana Svitková, Eva Barbušinová, Tomáš Derka, Monika Sláviková, Boris Klempa

**Affiliations:** 1 Institute of Virology, Biomedical Research Center, Slovak Academy of Sciences, Dúbravská cesta 9, 845 05 Bratislava, Slovakia; 2 Department of Microbiology and Virology, Faculty of Natural Sciences, Comenius University in Bratislava, Ilkovičova 6, 842 15 Bratislava, Slovakia; 3 Department of Biology and General Ecology, Technical University in Zvolen, T. G. Masaryka 24, 960 01 Zvolen, Slovakia; 4 Department of Ecosystem Biology, Faculty of Science, University of South Bohemia, Branišovská 1760, 370 05 České Budějovice, Czech Republic; 5 Department of Ecology, Faculty of Humanities and Natural Sciences, 17 Novembra č. 1, 081 16 Prešov, Slovakia; 6 Institute of Botany, Plant Science and Biodiversity Center, Slovak Academy of Sciences, Dúbravská cesta 9, 845 23 Bratislava, Slovakia; 7 Department of Breeding and Diseases of Game, Fish and Bees, Ecology and Cynology, University of Veterinary Medicine and Pharmacy in Košice, Komenského 73, 041 81 Košice, Slovakia; 8 Department of Ecology, Faculty of Natural Sciences, Comenius University in Bratislava, Iľkovičova 6, 842 15 Bratislava, Slovakia

**Keywords:** *Aedes* invasive mosquitoes, Asian bush mosquito, Culicidae, * Hulecoeteomyia japonica*, *Aedes japonicus japonicus*, Alien species, Central Europe

## Abstract

**Background:**

Invasive mosquitoes of the genus *Aedes* are quickly spreading around the world. The presence of these alien species is concerning for both their impact on the native biodiversity and their high vector competence. The surveillance of *Aedes* invasive mosquito (AIM) species is one of the most important steps in vector-borne disease control and prevention.

**Methods:**

In 2020, the monitoring of AIM species was conducted in five areas (Bratislava, Zvolen, Banská Bystrica, Prešov, Košice) of Slovakia. The sites were located at points of entry (border crossings with Austria and Hungary) and in the urban and rural zones of cities and their surroundings. Ovitraps were used at the majority of sites as a standard method of monitoring. The collected specimens were identified morphologically, with subsequent molecular identification by conventional PCR (*cox*1) and Sanger sequencing. The phylogenetic relatedness of the obtained sequences was inferred by the maximum likelihood (ML) method. The nucleotide heterogeneity of the Slovak sequences was analysed by the index of disparity.

**Results:**

A bush mosquito, *Aedes japonicus japonicus*, was found and confirmed by molecular methods in three geographically distant areas of Slovakia—Bratislava, Zvolen and Prešov. The presence of AIM species is also likely in Košice; however, the material was not subjected to molecular identification. The nucleotide sequences of some Slovak strains confirm their significant heterogeneity. They were placed in several clusters on the ML phylogenetic tree. Moreover, *Ae. j. japonicus* was discovered in regions of Slovakia that are not close to a point of entry, where the mosquitoes could find favourable habitats in dendrothelms in city parks or forests.

**Conclusion:**

Despite being a first record of the *Ae. j. japonicus* in Slovakia, our study indicates that the established populations already exist across the country, underlining the urgent need for intensified surveillance of AIM species as well as mosquito-borne pathogens.

**Graphical Abstract:**

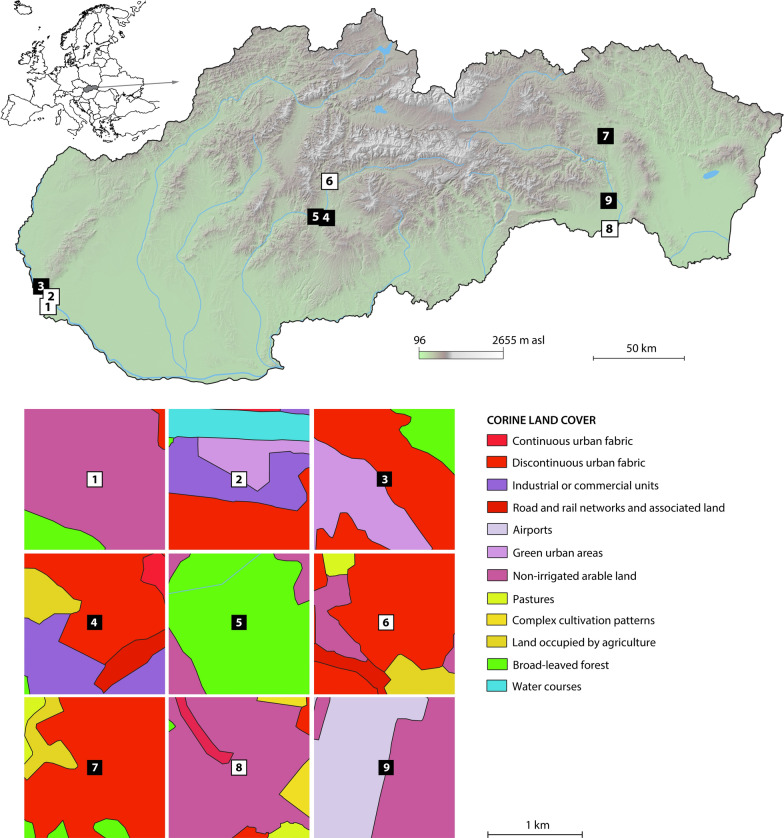

## Background

In the era of the re-emerging vector-borne diseases, the expansion and establishment of *Aedes* invasive mosquitoes (AIMs) as competent vectors of arboviral and filarial infections represent threats to human and animal health. Since their first introduction to Europe in the late 1970s, these AIMs have been considered responsible for several disease outbreaks in Europe, such as chikungunya in Italy and dengue in France [1, 2].

Among AIMs, the Asian bush mosquito *Aedes japonicus* (subgenus *Hulecoeteomyia*) (Theobald, 1901) originates from countries and islands in East Asia, such as China, Japan, Korea, Russia and Taiwan [3, 4]. The species is composed of four genetically distinct but morphologically nearly identical subspecies: *Ae. j. shintienensis* Tsai & Lien, 1950, *Ae. j. yaeyamensis* Tanaka, Mizusawa and Saugstad, 1979, *Ae. j. amamiensis* Tanaka, Mizusawa and Saugstad, 1979 and *Ae. j. japonicus* (Theobald, 1901) [5]. The last, *Ae. j. japonicus*, was introduced to North America through imported tyres in the 1990s and began to invade the East Coast. Currently, it is widely distributed in the eastern USA and Canada [6–8].

In Europe, the presence of *Ae. j. japonicus* was recorded for the first time by a tyre trading company in Montsecret, France, in 2000 [9]. Since then, this mosquito subspecies has invaded Europe and has already been recorded in Belgium, the Netherlands, Switzerland, Germany, Luxemburg, Slovenia, Croatia, Bosnia and Herzegovina, Serbia, Italy, Spain, Austria and Hungary [3, 4, 10].

In its native regions, *Ae. j. japonicus* inhabits mostly rock pools and tree holes, while in newly occupied areas it colonises mainly a wide range of artificial containers, which are well represented in urban environments. Larvae can be found in tyres, plastic or stone containers, buckets and stormwater drainage, among other locations. Regardless, *Ae. j. japonicus* favours more forested and shaded areas. This is not a strict preference and can be site-dependent [8, 11]. The subspecies is cold-tolerant and capable of dwelling in temperate regions, but larval rearing is limited to temperatures above 34–40 °C [8]. In European countries (e.g. Belgium), seasonal activity starts in May and lasts until October, with a peak between June and August [12].

*Aedes j. japonicus* is a mammophilic and ornithophilic species, and it is not considered a primary nuisance species to humans. This blood-feeding pattern has also been confirmed in Europe. This mosquito subspecies feeds on a wide range of hosts, with approximately 36% of hosts consisting of humans and 59% consisting of birds. Blood-meal analyses suggest that *Ae. j. japonicus* can serve as a bridge vector for pathogens from avian hosts to humans [3].

Concerns about the presence of *Ae. j. japonicus* are not limited to its rapid expansion through continents. *Aedes j. japonicus* is a competent vector of several arboviruses and even filarial nematodes. In Asia, the mosquito was identified as a vector responsible for outbreaks of Japanese encephalitis (JE). Natural infection by West Nile virus (WNV) and La Crosse encephalitis virus (LAC) was detected in wild-captured mosquitoes in the United States [8]. Moreover, infections by chikungunya virus (CHIKV), dengue virus (DENV) and Zika virus (ZIKV), and the zoonotic filarial nematodes *Dirofilaria repens* Railliet & Henry, 1911 and *Dirofilaria immitis* (Leidy, 1856) were successfully demonstrated under experimental conditions [13–15].

In Slovakia, only one invasive mosquito species, *Aedes albopictus* (Skuse, 1894), has been recorded so far. Four females were collected in the eastern part of the country in 2012 [16]. Despite repeated sampling in the area of the first collection, no other specimens of *Ae. albopictus* were trapped to the end of monitoring in this area in 2018 (unpublished).

In the current paper, we describe the first results of target surveillance of *Aedes* invasive species in Slovakia.

## Methods

The monitoring of invasive *Aedes* species was conducted in five areas of Slovakia from the end of July to the middle of October 2020. Possible points of entry (areas close to borders, highways and airports) and favourable areas for species expansion (urban and rural environment) were selected for our observations.

### Study area

**Fig. 1:**
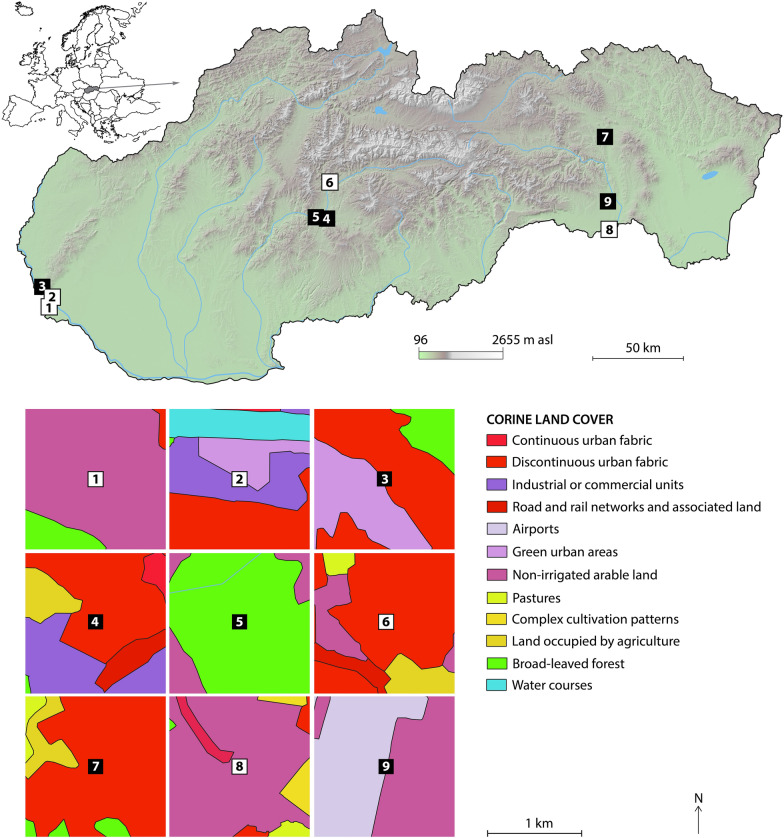
Position of monitoring areas in Slovakia (upper part) and Corine land cover types of the sampling sites (lower part). The sampling sites are labelled as follows: 1—Jarovce, 2—Janko Kráľ city park, 3—Patrónka (Bratislava), 4—TUZVO, 5—Stráže (Zvolen), 6—Sásová (Banská Bystrica), 7—Kolmanka city park (Prešov), 8—Milhosť and 9—Šebastovce I and II (Košice). Sites at which the presence or absence of *A. j. japonicus* was recorded/supposed are labelled with black and white squares, respectively

The monitoring areas were established in five larger cities covering a relatively broad range of conditions—Bratislava Zvolen, Banská Bystrica, Prešov and Košice (Fig. 1). The capital city of Slovakia, Bratislava, is located in the southwestern part of the country, and the population size is approximately 430,000 inhabitants [17]. Bratislava lies directly on the borders with Austria and Hungary and approximately 60 km from the border crossing to the Czech Republic. All three states are connected by the D2 motorway, which passes through Bratislava. The first monitoring site was at a gas station close to the border crossing at Jarovce (Grenzübergang Kittsee [AUT]—Jarovce [SK]), with a large rest area for trucks. The Janko Kráľ city park (42 ha) was established as the second monitoring site in Bratislava.

The last trapping site was in an industrial region of the city, Patrónka. This zone is covered mainly by business parks and industrial companies, and it is surrounded by a fragment of oak-hornbeam forest of 75 ha in size [18].

Zvolen is located in central Slovakia and has approximately 42,000 inhabitants. The first site represents an urban environment and was situated on the campus of the Technical University in Zvolen (TUZVO). The second site was located in a more natural, largely forested area, Stráže, in the vicinity of the R1 road.

Banská Bystrica (~ 78,000 inhabitants) is situated approximately 20 km north of Zvolen. Mosquito monitoring was performed at one site located within a housing estate bordering a rural landscape.

Mosquito sampling in northeastern Slovakia, in Prešov, was conducted along the watercourses and walls of old buildings in Kolmanka city park (see [19]). The population size of Prešov is approximately 89,000 inhabitants [17].

Košice (~ 240,000 inhabitants) is situated in southeastern Slovakia and is the second largest city in the country [17]. The first site, the Šebastovce, has a predominantly rural character and is close to Košice International Airport. The second site, Milhosť, is situated in the outskirts of Košice, and traps were set up at the border crossing with Hungary—Milhosť (Tornyosnémeti [HU]—Milhosť [SVK]) in the area surrounding Košice.

Corine land cover types [20] were used to visualise the type of environment in the vicinity of the sampling sites. The average daily climatic variables for the sampling areas were obtained from the nearest meteorological stations from the portal www.meteoblue.com.

### Mosquito collection

**Fig. 2:**
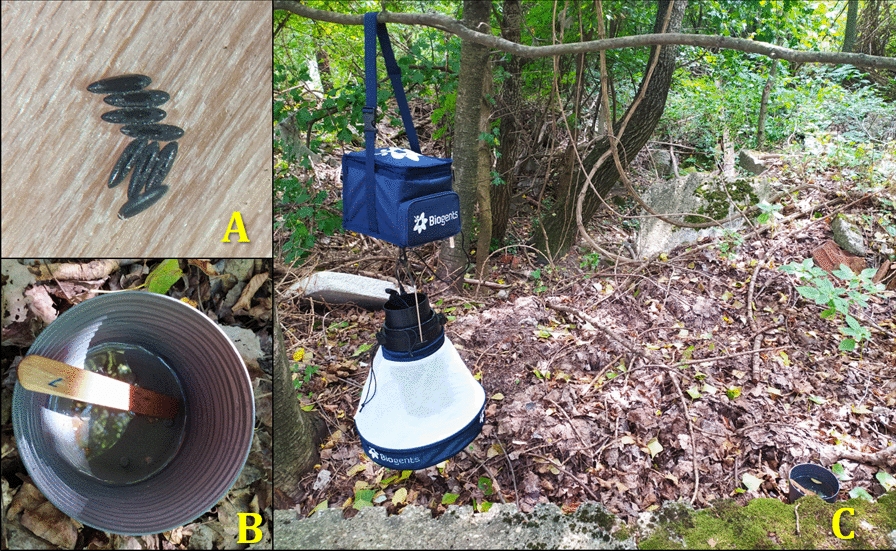
The surveillance of invasive *Aedes* mosquitos was based mainly on egg monitoring. Legend: Adult females laid eggs on a wooden tongue depressor (**a**) immersed in a plastic bucket filled with water (**b**). Image (**c**) shows an example of the habitat, a shaded place at the edge of an oak-hornbeam forest, where *Aedes japonicus japonicus* was collected

Oviposition traps (ovitraps), consisting of a dark-coloured plastic bucket (1 L) filled two-thirds full with tap water and a wooden tongue depressor immersed in the bucket, were used as a substrate for the oviposition of invasive *Aedes* species, as suggested by the European Centre for Disease Prevention and Control (ECDC) 2012 [11] long-term monitoring protocol and by AIM COST Action [21] (Fig. 2). Five ovitraps were placed per site (*n* = 25) in a standardised sampling scheme at a distance of 50–100 m from each other. Each ovitrap was checked biweekly for the presence of mosquito eggs and/or larvae. In the case of a positive finding, the wooden tongue depressor and/or the larvae were stored in 60% ethanol and were delivered for further processing to the Institute of Virology, Biomedical Center of Slovak Academy of Sciences. Additional trapping methods were used in some places. A BG-Pro trap (Biogents, Regensburg, Germany) enriched with dry ice was placed in close proximity to ovitraps in Patrónka, Bratislava, whereas a 100-l mesocosm was situated in the campus of the Technical University in Zvolen. The mesocosm was filled with stream water and supplied with 400 g of dry invasive goldenrod (*Solidago*) leaf litter.

In Prešov and Stráže, long-term monitoring was not possible, and mosquito sampling was conducted by sweep-netting. In Prešov, sweep-netting was performed between the 23rd and 28th of August 2020. In Stráže, the collection was performed only once, on the 11th of October 2020.

### Mosquito identification

All material delivered to the Institute of Virology was examined under a Leica EZ4 stereomicroscope (Leica Microsystems, Wetzlar, Germany) and identified by morphological features according to the keys of Becker et al. [11, 22] and ECDC (2012). The specimens were photographed under a Zeiss Axio Zoom.V16 stereomicroscope (Zeiss, Oberkochen, Germany) with an attached Canon 5D Mark IV camera (Canon, Tokyo, Japan). Each stacked habitus photography was created by stacking 200 focal planes using the software Zerene Stacker. If a potential invasive species was identified, DNA was extracted using the commercial QIAamp DNA Mini Kit (Qiagen, Hilden, Germany) following the steps in the protocol. Prior to extraction, the material was homogenised with 180 µl ATL buffer (part of the kit) and a 5 mm stainless bead in a Qiagen TissueLyser II (Qiagen, Hilden, Germany) at 30 Hz/1 min.

Molecular identification was performed by conventional polymerase chain reaction (PCR) targeting a 700 base pair (bp)-long fragment of the cytochrome *c* oxidase subunit I (*cox*1) gene, employing the primer pair MTFN/MTRN designed for mosquito barcoding [23]. The products were visualised on 1.5% agarose gel. Positive amplicons were cleaned up by ExoSAP-IT™ (Applied Biosystems, Waltham, MA, USA) and sequenced in both directions by Sanger sequencing in a commercial laboratory (Microsynth, Vienna, Austria).

The obtained sequences were edited by MEGA 7 and compared by the Basic Local Alignment Search Tool (BLAST) with data available in GenBank. The genetic relatedness of the Slovak material to European strains deposited in the GenBank database was examined by the maximum likelihood (ML) method employing the Tamura-Nei model-implemented gamma distribution with invariant sites (T92 + GI). The model was tested by a bootstrap resampling of 50 replicates. The disparity index test was calculated for all Slovak sequences. The homogeneity of the disparity index pattern was estimated by a Monte Carlo test with 500 replicates. *P*-values of less than 0.05 were considered significant (MEGA 7).

## Results

**Fig. 3:**
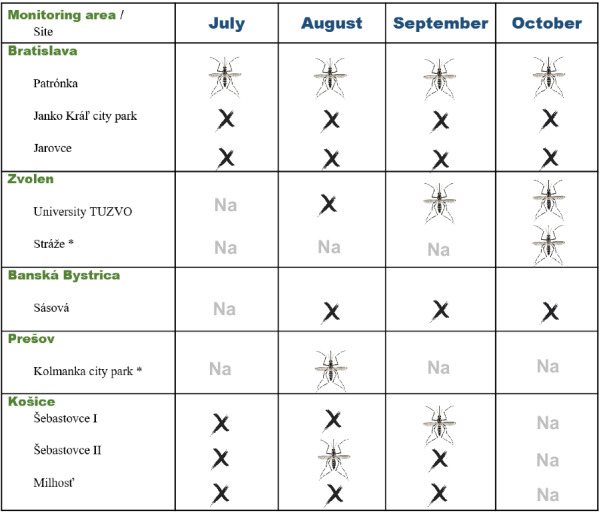
Seasonal records of *Aedes japonicus japonicus* in the monitored area in Slovakia, 2020. Asterisk indicates sweep-netting, mosquito—positive, x—negative, na—not applicable. The finding in Košice is recorded as “suspected *Aedes* invasive species” because molecular confirmation was not performed

The invasive mosquito *Ae. j. japonicus* was trapped in three monitored cities of Slovakia: Bratislava, Zvolen and Prešov. The occurrence of this AIM is also suspected in Košice (Fig. 3).

Eggs and *Aedes* larvae were present in one sampling site of Bratislava, in the Patrónka industrial zone (Fig. 1). The first oviposition was observed in late July, and some eggs had already hatched into larvae. In total, eggs were detected in Patrónka five times during the whole monitoring season (Fig. 3). The egg clutch size on the tongue depressors varied between 2 and 108 eggs, with the highest number found on the 18th of August 2020. A BG-Pro CO_2_ trap was placed in close proximity to the ovitraps but no adult *Ae. j. japonicus* were caught in Patrónka, Bratislava.

The sampling season in the urban area at TUZVO was initiated at the beginning of August, and the first hatched eggs and fourth-instar *Aedes* larvae were found on the 23rd of September 2020. In total, eggs were observed in that area during three sampling periods. The maximum number of eggs counted on a tongue depressor at TUZVO was 260 eggs, collected on the 23rd of September, and a minimum of nine eggs were collected in the middle of October 2020. In addition, newly hatched adult females were collected from a mesocosm on the TUZVO campus on the 29th of September 2020. The water temperature in the mesocosm ranged from 9.4 to 22.6 °C (15.8 °C on average), the pH ranged from 7.1 to 7.7 (7.5), and the aquatic environment was rich in nutrients (dissolved organic carbon = 41.7 mg/l, dissolved organic nitrogen = 2.1 mg/l, soluble reactive phosphorus = 27.8 µg/l). At the second sampling site, in Zvolen, Stráže, monitoring was performed just once. Adults of *Ae. j. japonicus* (*n* = 4) were caught by sweep-netting on the 11th of October 2020.

No invasive mosquito species catches were reported from the neighbouring city, Banská Bystrica. In the city of Prešov, 24 adults of *Ae. j. japonicus* were trapped in August.

Eggs were also found in the southeastern part of Slovakia in the city of Košice in both traps at the Šebastovce sampling sites (Šebastovce I and II). The first egg hatch was observed in August, and eggs were also subsequently found in September 2020. Invasive mosquito species were not detected at the site located at the border crossing Hungary at Milhosť.

**Fig. 4:**
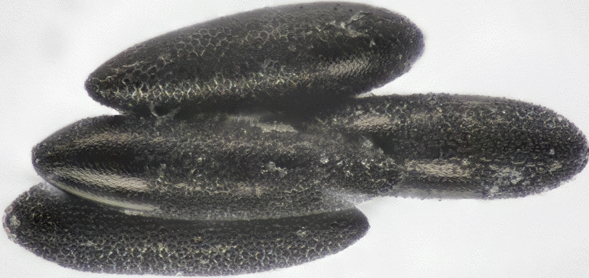
*Aedes japonicus japonicus* eggs with the specific abrasive pattern on the exochorion

*Aedes* mosquitoes at different developmental stages (eggs, larvae and/or adults) were found in the four monitoring areas of Slovakia. The eggs on the tongue depressors were laid separately and were oval in shape. All examined eggs had the specific abrasive pattern on the exochorion, which was the main determinant for further processing (Fig. 4). The only native species with a similar surface occurring in Slovakia is *Aedes geniculatus* (subgenus *Dahliana*) (Olivier, 1791) [24]. Hence, all morphologically identified eggs were classified as “*suspected invasive species*”. Two samples of eggs from a tongue depressor found in Košice were not delivered for morphological and molecular determination and were identified only as suspected invasive species.

The fourth-instar larvae were identified as *Ae. japonicus* sensu lato according to the main morphological determinants, i.e. the arrangement of the head cephalic setae (C)—5C and 6C and a single-branched subventral tuft inserted between pecten teeth [11].

Adult mosquitoes were stored in 60% ethanol, and for this reason was their identification problematic. The adults trapped at the Stráže and TUZVO sites, Zvolen, were successfully morphologically identified as *Ae. japonicus* s.l. The material from Prešov was morphologically identified as *Aedes koreicus* (subgenus *Hulecoeteomyia*) (Edwards, 1917) (see [19]), although further molecular analyses (described below) showed a higher genetic identity with *Ae. j. japonicus* than with *Ae. koreicus*.

Altogether, 16 samples were processed by molecular methods. Regrettably, larval DNA (*n* = 2) was not successfully extracted, and conventional PCR targeting the *cox*1 fragment selected 11 egg and three adult samples for further Sanger sequencing. After the initial BLAST analyses of the obtained nucleotide sequences, one egg sample from Patrónka, Bratislava, was determined to be genetically related to *Ae. geniculatus* (MT993491). Finally, molecular identification revealed *Ae. j. japonicus* in 13 egg samples. The nucleotide sequences confirmed 99.78–100% identity to several strains of *Ae. j. japonicus* deposited in GenBank (e.g. MN103383, MN058619, MN058630, MN058628).

**Fig. 5:**
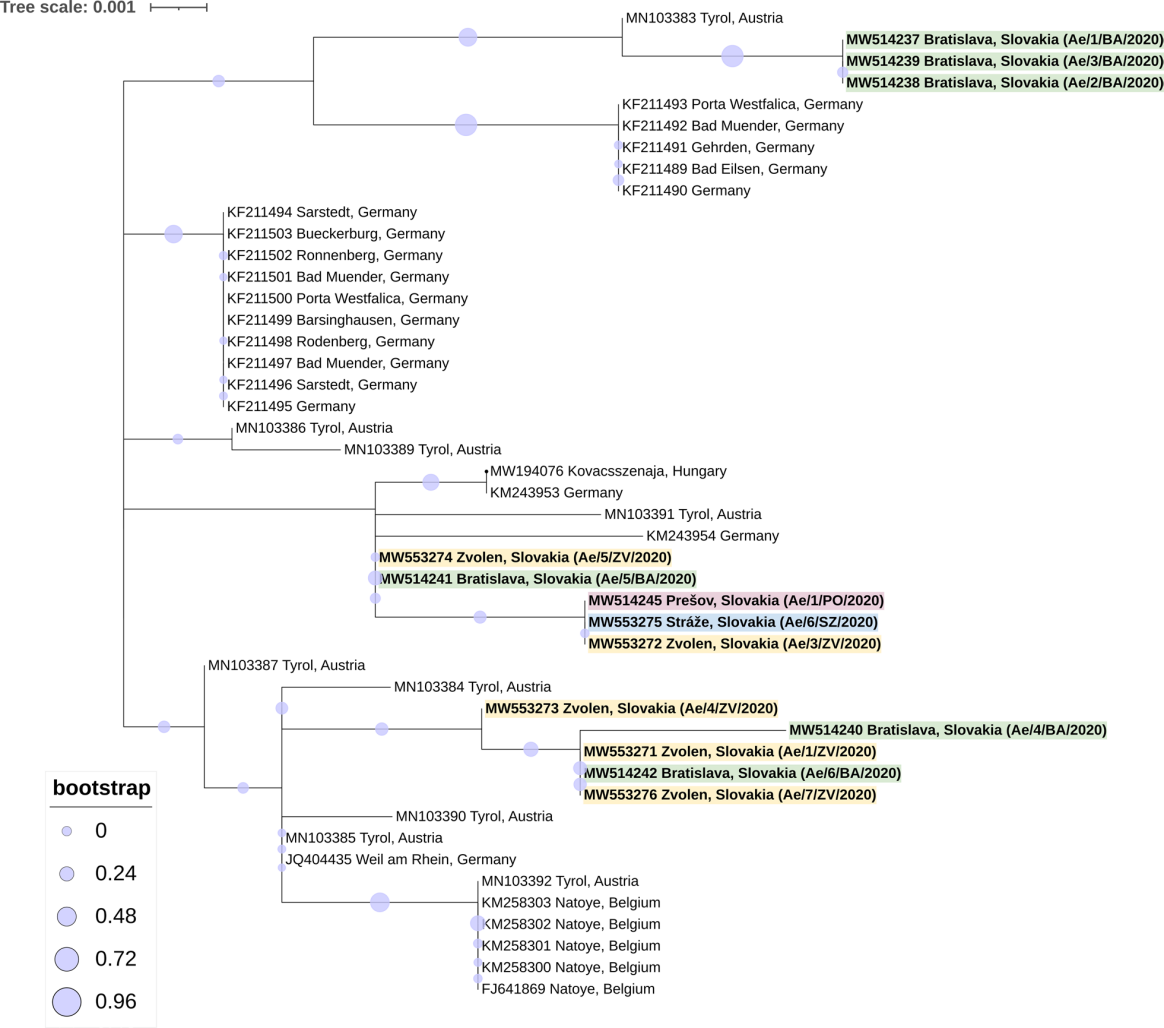
The phylogenetic relatedness of *Aedes japonicus japonicus* collected in Slovakia, 2020. Legend: The maximum likelihood phylogenetic tree based on the *cox*1 fragment (700 bp) showing the relatedness of Slovak strains (bold) to nucleotide sequences obtained from *Ae. j. japonicus* collected in other European countries. The different monitored areas are highlighted in different colours—Bratislava (green), Zvolen (yellow), Stráže (blue), Prešov (pink)

The ML model based on partial *cox*1 sequences of *Ae. j. japonicus* indicated genetic differences between Slovak strains, even among the isolates collected from the same location (Fig. 5). Significantly different nucleotide frequencies (*P* = 0.01) were estimated between the four Slovak *Ae. j. japonicus* strains (Ae/4/ZV/2020–Ae/3/ZV/2020; Ae/6/SZ/2020; Ae/1/PO/2020) (Table 1).

The nucleotide sequences of *Ae. j. japonicus* obtained in this study are available under the GenBank accession numbers MW514237–MW51422, MW514245, MW553271–MW553276.

## Discussion

Since its first introduction to Europe in the late 1970s, *Ae. j. japonicus* subspecies has invaded several countries on the continent [1, 2]. Among the countries neighbouring Slovakia, *Ae. j. japonicus* occurrence has been reported in Austria and Hungary and was detected in both countries for the first time in 2012 [25, 26]. Currently, this mosquito creates well-established populations in those countries and occurs in highly populated areas such as Vienna and the Lake Balaton recreation area in Hungary [27, 28].

It is not surprising that this alien species was also detected in Slovakia. We assumed that due to the close socioeconomic relationships with our neighbours, the border crossing in the southwestern part of the country near Bratislava would be the primary point of entry. However, no positive observations were made in close proximity to the Slovak-Austrian border. *Aedes j. japonicus* was found in Bratislava, but it was found much farther into the metropolitan area, in the industrial zone of Patrónka.

Although several field studies focused on adult mosquitoes were conducted from 2010 to 2013 and from 2015 and 2017 in the same areas as those sampled in the present study, *Ae. j. japonicus* has not been collected in Slovakia until now (e.g. [29, 30]). According to Jansen et al. [4], adult *Ae. j. japonicus* are trapped only in areas with high population densities, and for this reason, adult traps are not the best monitoring approach. An adequate sampling method and thoughtful site selection in favourable environments are necessary to discover this species in Slovakia. For example, Patrónka, Bratislava, is surrounded by a fragment of oak-hornbeam forest that creates a shaded area with numerous natural or artificial sites that are suitable for *Ae. j. japonicus* breeding. A very similar habitat was observed in Kolmanka park, Prešov, where tree holes (dendrothelms) create suitable conditions for *Ae. j. japonicus* oviposition. *Aedes j. japonicus* is also capable of occupying relatively high elevations of the country, such as in Zvolen and Stráže in central Slovakia (approximately 287 and 316 m a.s.l.). In Zvolen, the mosquito was detected in an urban area, while the Stráže site represents a more natural habitat surrounded by a broadleaf forest.

The common problem in *Ae. j. japonicus* identification is the possible misidentification of the adult *Ae. koreicus*. Several discriminating morphological features (e.g. the subbasal dark band on the hind femurs and male genitalia) largely overlap between these species. Furthermore, it is assumed that both species are much more genetically related than was previously expected. *Aedes koreicus* creates a monophyletic group together with all subspecies of *Ae. japonicus* s.l. [5]. We encountered this problem in our study when an adult specimen collected in Kolmanka, Prešov, was incorrectly identified as *Ae. koreicus* [19]. Further molecular analyses showed that the nucleotide sequences of the individual from Prešov shared a higher genetic identity with *Ae. j. japonicus* than with *Ae. koreicus*. The molecular approach seems to be necessary to provide the correct identification of invasive *Aedes* species, especially for the differentiation between *Ae. koreicus* and *Ae. japonicus* s.l. In Slovakia, the co-occurrence of both species is plausible. Like the bush mosquito, *Ae. koreicus* was detected in Hungary and Austria [31, 32].

The selected molecular method employing a primer pair originally designed for mosquito barcoding [23] can be successfully used for species identification. However, the chosen fragment of the *cox*1 gene does not seem to be an adequate determinant for phylogenetic analyses. The tree did not show any strong and/or unique ancestor group to confirm the origin of *Ae. j. japonicus* collected in Slovakia. Previously described genetic studies performed on European strains support the assumption of at least two separate introductions (genotypes I and II) of *Ae. j. japonicus* to the continent [33, 34]. A better understanding of the origin of *Ae. j. japonicus* in Europe can be acquired by microsatellite genotyping based on the dehydrogenase subunit 4 (*nad*4) gene. *Aedes japonicus* alone consists of 45 different haplotypes, among which some have already been revealed in Europe within genotypes I and II (H1, H5, H6, H9, H10, H33, H44, H45), and the co-occurrence of the haplotypes in the same area is not uncommon [33, 34]. Although there are some known differences between several Slovak strains, our knowledge of their genetic polymorphism is currently limited, and complex microsatellite analyses should be implemented in future studies.

The fact that this invasive subspecies was found in different regions of Slovakia, even those that are not in proximity to an unambiguous point of entry (e.g. Prešov), is alarming. It seems that our report does not point out a single case of penetration but rather reveals established populations across the country. Additionally, genetic analyses show variability among Slovak nucleotide sequences, which may suggest multiple introductions of *Ae. j. japonicus* to the country.

Although this mosquito is not considered a major vector of viral and parasitic diseases, it is able to transmit a broad range of pathogens [35]. The greatest concern for newly inhabited areas relates to the potential spread of re-emerging mosquito-borne viruses, such as WNV, CHIKV or DENV. Several mosquito-borne pathogens, such as WNV, Usutu virus and *Dirofilaria* parasites, are quite abundant in Slovakia [30, 36, 37]. This study confirms the urgent need for long-term, country-wide and targeted surveillance of AIMs intertwined with intensified surveillance of mosquito-borne pathogens.

**Table 1:**
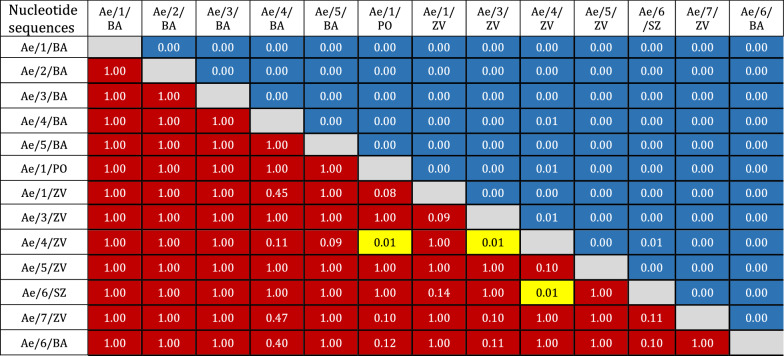
Homogeneity of the disparity index estimated among sequences obtained from *Aedes japonicus japonicus* collected in Slovakia, 2020

*P-*values estimated by the Monte Carlo test are shown below the diagonal. Significant *P*-values (less than 0.05) are highlighted in yellow. The estimates of the disparity index per nucleotide site are shown above the diagonal

## Data Availability

The majority of the data are included in the present manuscript. The nucleotide sequences are available under accession nos. MW514237–MW51422, MW514245 and MW553271–MW553276 in GenBank.

## Abbreviations

CHIKV: Chikungunya virus
DENV: Dengue virus
JE: Japanese encephalitis
WNV: West Nile virus
LAC: La Crosse virus
ZIKV: Zika virus
ECDC: European Centre for Disease Prevention and Control
AIM: *Aedes* invasive mosquito
DNA: Deoxyribonucleic acid
Bp: Base pair
*cox*1: Cytochrome *c* oxidase subunit I
PCR: Polymerase chain reaction
ML: Maximum likelihood
T92 + GI: Tamura-Nei model with gamma distribution with invariant sites
BLAST: Basic Local Alignment Search Tool
*nad*4: Dehydrogenase subunit 4
H: Haplotype
